# Evaluation of the significance of complement-related genes mutations in atypical postinfectious glomerulonephritis: a pilot study

**DOI:** 10.1007/s11255-023-03831-7

**Published:** 2023-10-17

**Authors:** Feng Xu, Changming Zhang, Mingchao Zhang, Xiaodong Zhu, Shuiqin Cheng, Zhen Cheng, Caihong Zeng, Song Jiang

**Affiliations:** National Clinical Research Center for Kidney Disease, Jinling Hospital, Nanjing Medical University, 305 East Zhongshan Road, Nanjing, 210018 Jiangsu China

**Keywords:** Postinfectious glomerulonephritis, Hypocomplementemia, Complement-related gene, Mutation

## Abstract

**Background:**

Postinfectious glomerulonephritis with C3-dominant glomerular deposition (C3-PIGN) involves C3-dominant glomerular deposition without immunoglobulin. Atypical C3-PIGN involves persistent hypocomplementemia. We investigated the clinical features and explored complement-related gene mutations in atypical PIGN patients.

**Methods:**

We enrolled atypical C3-PIGN patients and collected data regarding the clinical presentation and pathological characteristics and follow-up data. We measured the levels of complement associated antibodies and performed whole-exome sequencing (WES) to detect mutations in complement-related genes.

**Results:**

The analysis included six atypical C3-PIGN patients. All patients were antistreptolysin-O (ASO) positive. All patients had varying degrees of hematuria, and four patients had proteinuria. None of the patients were positive for complement-related antibodies. All patients possessed mutations of genes related to the complement pathway, including alternative complement pathway genes—CFI, CFH, CFHR3, CFHR5; the lectin pathway gene—MASP2; and the common complement pathway gene—C8A. The rare variant of CFHR3 has been reported in C3 glomerulonephritis. During 56–73 months of follow-up, the levels of urine markers in three patients recovered within 6 months, and the remaining patients had abnormal urine test results over 12 months. Patients who received glucocorticoid therapy recovered faster.

**Conclusions:**

Our study suggested that complement-related gene mutations may be an important cause of persistent hypocomplementemia in atypical C3-PIGN patients. In addition to variations in alternate pathway-related genes, we also found variations in lectin pathway-related genes, especially *MASP2* genes. Although the overall prognosis was good, atypical C3-PIGN patients exhibited a longer period for recovery. Our results suggested that atypical C3-PIGN patients should receive more medical attention and need testing for mutations in complement-related genes.

## Introduction

Acute postinfectious glomerulonephritis (PIGN) is a complication of streptococcal and other infections and can occur in both children and adults [1, 2]. The primary clinical manifestation of PIGN is acute nephritis syndrome [3]. In PIGN, glomerular injury is mediated by a host immune response that is triggered by an infectious agent. The activation of complement plays a pivotal role in PIGN. Hypocomplementemia is an important feature of active PIGN, which is characterized by low serum levels of complement C3 and normal levels of C4, thus suggesting that the infection induces the activation of the complement system.

The primary histological manifestation of PIGN during the acute phase is proliferative lesions and exudative lesions in the glomerular capillaries, while the recovery period is associated with proliferative lesions in the glomerular mesangial cells. There are two PIGN immunophenotypes: one type is Ig + C3-PIGN, which involves glomerular deposition of immunoglobulin G (or IgA) and C3, while the other is C3-PIGN, which is mainly characterized by the glomerular deposition of C3, with or without weakly positive immunoglobulin results [4]. C3-PIGN exhibits similar histological and immunofluorescence characteristics to C3 glomerulonephritis (C3GN).

Most patients with C3-PIGN have an excellent prognosis. In the majority of cases, hypocomplementemia resolves within 6–8 weeks; hematuria and proteinuria are likely to resolve within weeks or months. This trajectory is in stark contrast with the poor long-term outcomes of C3GN patients, which often include persistent hypocomplementemia, proteinuria and hematuria. It is currently believed that the complement system in patients with C3GN is abnormally regulated and that this pathology is very difficult to correct. However, long-term follow-up observations have shown that few patients with C3-PIGN are affected by persistent hypocomplementemia and kidney damage [5–8]. This is an atypical form of C3-PIGN and is very difficult to distinguish from C3GN [9].

Previous studies indicate that C3GN patients have various complement-related gene mutations, including mutations in the C3, membrane cofactor protein (MCP), thrombomodulin (THBD), complement factor H (CFH), complement factor I (CFI), complement factor B (CFB) and complement factor H-related proteins (CFHR) genes [10–12]. These mutated genes caused abnormal regulation of the complement system. Due to the similar clinical and pathological features between C3GN and atypical C3-PIGN patients, we hypothesize that atypical C3-PIGN patients may carry mutations in complement-related genes. In this study, we investigated the clinicopathological characteristics and long-term prognosis of atypical C3-PIGN patients. In addition, we performed whole exon sequencing (WES) to identify the mutations in all genes related to the complement pathway.

## Materials and methods

### Patients

A total of 74 patients with C3-PIGN who had been registered in the Nanjing Glomerulonephritis Registry at the National Clinical Research Center of Kidney Diseases, Jinling Hospital, from January 2010 to December 2018 were included in this study using the diagnostic criteria as follows: (1) history of preceding infection; (2) hematuria with or without proteinuria(> 0.4 g/24 h); (3) decreased serum C3 level; (4) characteristics of postinfectious glomerulonephritis—(a) proliferative glomerulonephritis on light microscopy (LM); (b) C3 deposition without immunoglobulin and C1q on immunofluorescence (IF) microscopy; (c) ‘hump-like’ subepithelial electron-dense deposits on electron microscopy (EM).

The atypical C3-PIGN was identified as C3-PIGN combined with hypocomplementemia lasting more than 12 months. Six patients were diagnosed with atypical C3-PIGN (Supplementary Fig. 1). We collected a range of baseline clinical and pathological data, measured the levels of complement-related antibodies and performed WES. All patients provided informed written consent. The study adhered to the guidelines of the Declaration of Helsinki and was approved by the Local Ethics Committee of National Clinical Research Center for Kidney Diseases, Jinling Hospital (2022DZKY-060-01, Nanjing, China).

All 6 atypical C3-PIGN patients were followed up regularly. All patients were followed-up at 1, 3, 6 and 12 months after discharge and twice a year after 12 months. The follow-up time ranged from 56 to 73 months.

### Renal pathological changes

The renal biopsy tissues were examined by light microsope (LM), Immunofluorescence (IF) and electron microscope (EM). The paraffin-embedded tissue sections were stained with hematoxylin–eosin (HE), periodic acid-Schiff (PAS), methenamine silver, and Masson trichrome. A direct immunofluorescence method was used to detect IgG, IgA, IgM, C3c, C4c, C1q, mannan-binding lectin associated serine protease-2 (MASP2) and fibrin expression. The EM tissue was first fixed with 3.75% cold glutaraldehyde, then fixed with 1% osmium tetroxide and embedded in resin. Ultrathin sections were cut at 70–80 nm, double stained with uranium acetate and lead citrate, and examined with a Hitachi 7500 transmission electron microscope.

Renal biopsy specimens were examined by two renal pathologists. The pathological score of each patient's kidney biopsy specimen was determined. According to the pathological classification criteria, acute tubular injury (ATI) and interstitial fibrosis and renal tubular atrophy (IFTA) were defined based on the percentage of lesions in the cortical area. Crescents were described according to the number of lesions. Immunofluorescence intensity was semiquantitatively graded as follows: −, trace, 1 + , 2 + , and 3 + .

### Complement and complement-related antibody testing

Serum C3 and C4 levels were measured by nephelometry (Beckman Coulter, California, USA). C3 nephritic factor (C3NeF) level was evaluated by hemolytic assays using preactivated sheep erythrocytes [13, 14], and the presence of Factor H autoantibody was detected by Enzyme Linked Immunosorbent Assay (ELISA) [15, 16]. Normal ranges were defined as follows: C3, 80–180 mg/dl [mean ± 2 standard deviations (SD)]; C4, 10–40 mg/dl (mean ± 2 SD). All functional assays were repeated three times.

### Whole-exome sequencing

#### Library preparations and sequencing

Genomic DNA was extracted from patients using a DNeasy Blood & Tissue Kit (QIAGEN). Exome capture was performed using SureSelect Human All Exon V6 (Agilent Technologies) according to the manufacturer’s instructions. The concentration of the libraries was measured by Qubit® 2.0 Fluoromete. The quality and size of the libraries were measured by a 2100 Bioanalyzer High Sensitivity DNA Assay according to the reagent kit guide. For Illumina sequencing, the qualified libraries were subjected to 2 × 150 bp paired-end sequencing on the Illumina HiSeq X-ten platform (Illumina).

### Variant calling and annotation

Variants [single-nucleotide variants (SNVs) and indels] were genotyped from recalibrated BAM files using the multisample processing mode of the Unified Genotyper tool from GATK. Then, VQSR (Variant Quality Score Recalibration) was used to reduce the number of false positives during variant calling. Copy number variation was identified by XHMM (eXome Hidden Markov Model) v1.0. SNVs and indels were annotated using ANNOVAR software against multiple databases, including HGVS variant description, population frequency, disease or phenotype and variant functional prediction. Screening for *CFH, MCP, CFI, CFB, C3* and *THBD* coding sequences was performed by amplicon-based next-generation sequencing [14]. Rare functional variants (missense, nonsense, indel, or splicing variants with minor allele frequency, MAF < 0.001 in 1000 Genomes and ExAC databases) were selected and defined as likely pathogenetic or pathogenetic when published functional studies were available.

We used SIFT, PolyPhen-2 and Mutation Taster to predict the functional effects of each missense and amino acid substitution. To predict the three-dimensional protein structure and the internal molecular segments, we constructed a mimetic human GDF3 protein by using the SWISS–MODEL (http://swissmodel.expasy.org) server.

## Results

### Clinical features of atypical C3-PIGN patients

The six patients included four males and two females; three patients were adults. Three of these patients had a history of prodromal infection in the upper respiratory tract, and the time from infection to the appearance of acute nephritis syndrome in each patient was 13 days, 14 days, and 18 days. The levels of serum creatinine (Scr) at renal biopsy ranged from 0.55 to 2.7 mg/dl. These patients had varying degrees of hematuria. Four patients were positive for proteinuria, and two patients experienced proteinuria > 1 g/24 h. All patients were ASO positive, one patient was antinuclear antibody (ANA) positive, and one patient was positive for the hepatitis B antigen. Three patients had hypertension, and all patients had a normal serum level of C4 (Table 1).

**Table 1.** Clinical and biochemical parameters of patients

Patients	Age (years)	Sex	Serum C3 (mg/dl)	Serum C4 (mg/dl)	URBC (10^4^/l)	UPro (g/24 h)	sCreat (mg/dl)	ASO IU/ml	CRP mg/L	Other
1	12	Male	23.9	19.6	400 dRBC	0.25	0.55	221	< 0.50	
2	51	Female	30	10	245 dRBC	5.53	2.7	158	6.3	ANA1:256
3	13	Male	59.2	25.4	137 dRBC	0.21	0.9	375	10.6	
4	26	Male	74.9	26.6	23 dRBC	1.83	0.93	415	0.8	HBsAg + ,HBeAb + ,HBcAb +
5	13	Male	47.6	10	2500 dRBC	0.9	0.63	636	0.1	
6	31	Female	18.3	9.87	20 dRBC	0.53	0.58	1870	2.6	

*URBC* urinary red blood cell, *UPro* 24-h urinary protein quantity, *Scr* serum creatinine, *dRBC* dysmorphic red blood cell, *Serum C3* normal range > 80 mg/dl, *Serum C4* normal range > 10 mg/dl, *ASO* Anti-Streptolysin O, *CRP* C-reactive protein

Three patients were treated with corticosteroids, and two of them additionally received angiotensin-converting enzyme inhibitor/angiotensin receptor blocker (ACEI/ARB) therapy. The other three patients were treated conservatively by renin–angiotensin system blockade alone and did not receive steroids or other forms of immunosuppressive therapy.

**Fig. 1:**
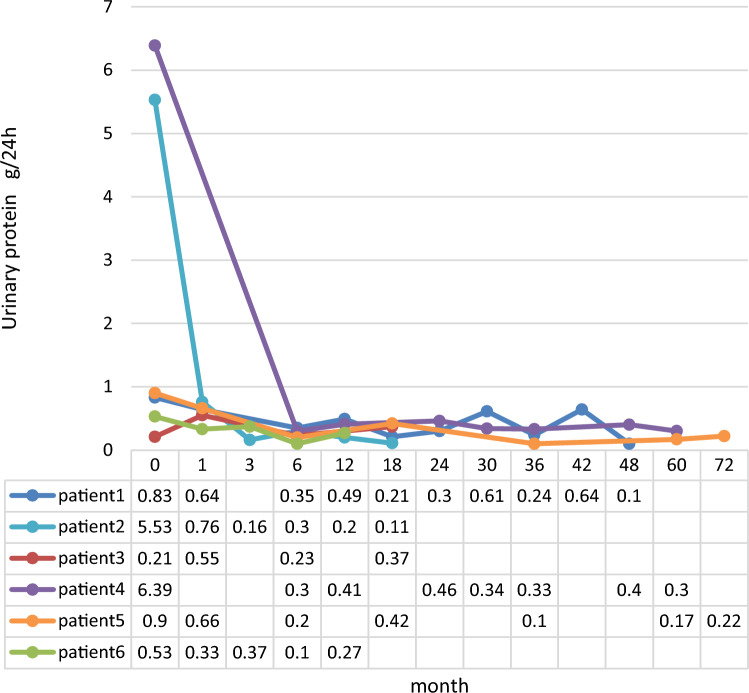
Follow up of 24 h urinary protein

The levels of urinary markers in three patients returned to normal levels within 6 months. However, the abnormal levels of urinary markers in patients 1, 4 and 5 returned to normal after 8 months, 18 months and 27 months, respectively (Fig. 1). In general, patients did well with no significant decline in renal function, both over the short and long terms. The serum creatinine levels in patient 2 returned to normal by the 6 month follow-up. Normal renal function was maintained in the other five patients.

**Fig. 2:**
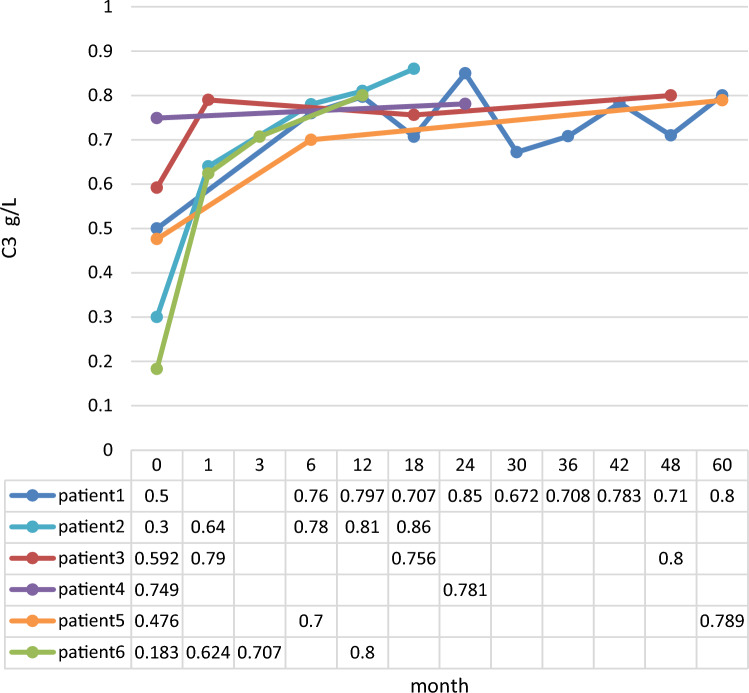
Follow up of serum C3

The plasma levels of C3 in 2 patients had recovered by 12 month follow-up. Four patients had hypocomplementemia, with a duration ranging from 12 to 60 months (Fig. 2). The plasma C3 level was normal in patient 1 after repeated testing at the 24-month follow-up but had become substantially reduced by the 30 month follow-up; this was complicated by proteinuria and microscopic hematuria.

### Pathological features of atypical C3-PIGN

**Fig. 3:**
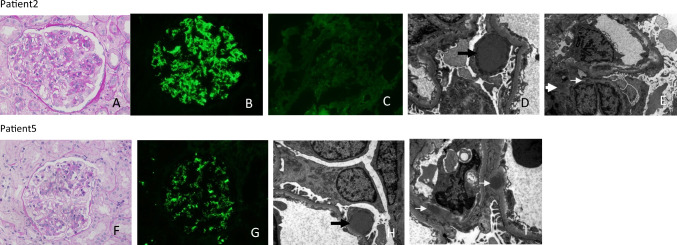
Light microscopy (LM), Immunofluorescence microscopy (IF) and electron microscopy (EM) in atypical C3-PIGN. **a** Shows a predominantly mesangial proliferative with segmental membranoproliferative glomerulonephritis (PAS 400 ×), **b** shows C3 deposited along the glomerular capillary and mesangial area (IF 400 ×), **c** shows C4 (trace) segmental deposited along the glomerular capillary and mesangial area (IF 400 ×), **d**–**e** EM showing subepithelial (thick black arrow), mesangial (thick white arrow)and subendothelial deposits (white arrow). **f** Shows a predominantly mesangial proliferative with segmental endocapillary proliferative glomerulonephritis (PAS 400 ×), **g** shows C3 deposited along the glomerular capillary and mesangial area (IF 400 ×), (**h**, **i**) EM showing subepithelial (thick black arrow) and intramembranous deposits (white arrow)

The pathological features of atypical C3-PIGN patients are shown in Table 2. Four patients exhibited mesangial proliferative glomerulonephritis; numerous infiltrating neutrophils were observed in the glomerular capillaries, 1 patient showed mesangial proliferation with segmental membranoproliferative glomerulonephritis, and the remaining patients had mesangial proliferative with segmental endocapillary proliferative glomerulonephritis. Immunofluorescence analysis further revealed C3 deposition in the mesangial area and the capillary wall in 5 patients; the other patient showed intense C3 (3 +) staining with mild IgA ( +) staining. EM revealed a hump-like subepithelium and electron dense deposits of mesangial cells in all patients. Four patients exhibited intramembranous deposits, and 3 patients had subendothelial deposits (Fig. 3).

**Table 2:** Pathological features of renal biopsy

Patients	Pattern of injury	total glomeruli	GS	Crescents	ATI%	IFTA%	IF microscopy	EM deposits
1	Mesangial proliferative GN	40	0	0	0	5	C3 (2 +), MASP2 (2 +)	SU, SE, IN, MES
2	Mesangial proliferative with segmental membrano proliferative GN	18	2	2	30	10	C3 (2 +), C4c ( ±), MASP2 ( +)	SU, SE, MES
3	Mesangial proliferative GN	44	0	0	0	0	C3 (2 +), MASP2 (2 +)	SU, IN, MES
4	Mesangial proliferative GN	48	2	5	15	5	C3 (3 +), IgA(1 +)	SU, IN, MES
5	Mesangial proliferative with segmental endocapillary proliferative GN	47	0	0	15	0	C3 (2 +)	SU, SE, IN, MES
6	Mesangial proliferative GN	22	0	0	10	0	C3 (2 +)	SU, MES

*ATI* acute tubular injury, *GN* glomerulonephritis, *GS* glomerulosclerosis, *IFTA* interstitial fibrosis and tubular atrophy, *IN* intramembranous, *MES* mesangial, *SE* subendothelial, *SU* subepithelial

### Complement-related antibody testing

We detected C3NeF, factor H, and anti-factor H in all patients, and none were positive.

### The identification of mutations in complement-related genes

Six patients possessed mutations in complement-related genes. Seven mutations were identified in four genes in the alternative complement pathway. We identified two missense mutations in the *CFH* gene (p. V837I and p. I551T), one mutation in the *CFHR3* gene (p. R142C), one mutation in the *CFHR5* gene (p. G145E) and three mutations in the *CFI* gene (p. R399H, p. R406H and p.R414H). We also identified two mutations (p. D371Y and p. H155R) in the *MASP2* gene, which is a gene that is involved in the lectin pathway, and one mutation (p. R485L) in *C8A*, which is a common gene that encodes the alpha subunit of C8 and participates in the formation of the membrane attack complex (Table 3).

**Table 3:** Rare coding variants in complement system genes

ID	Gender	Age of onset	Gene	Nucleotide change	Amino acid change	SIFT	Polyphen2	Mutation Taster	CADD	Clinical manifestations
1	M	12	*MASP2*	c.1111G > T	p.Asp371Tyr	D	P	P	14.12	Hematuria
2	F	51	*MASP2*	c.1111G > T	p.Asp371Tyr	D	P	P	14.12	Hematuria with proteinuria
			*C8A*	c.1454G > T	p.Arg485Leu	D	P	P	12.54	
3	M	13	*MASP2*	c.1111G > T	p.Asp371Tyr	D	P	P	14.12	Hematuria
			*CFH*	c.1652 T > C	p.Ile551Thr	T	D	N	13.01	
4	M	26	*MASP2*	c.1111G > T	p.Asp371Tyr	D	P	P	14.12	Hematuria with proteinuria
			*CFI*	c.1196G > A c.1217G > A c.1241G > A	p.Arg399His p.Arg406His p.Arg414His	T	P	P	2.089	
5	M	13	*MASP2*	c.464A > G	p.His155Arg	D	P	D	15.65	Hematuria with proteinuria
6	F	31	*CFH*	c.2509G > A	p.Val837Ile	T	B	D	0.822	Hematuria with proteinuria
			*CFHR5*	c.434G > A	p.Gly145Glu	T	B	P	0.011	
			*CFHR3*	c.424C > T	p.Arg142Cys	D	D	P	13.96	

*D* Damaging, *P* Probably damaging, *T* Tolerence, *B* Benign

All of these mutations had been reported previously and were heterozygous variants. In addition, all of these mutations were located in the coding region of their respective genes and were missense mutations. Polyphen-2, SIFT and MutationTaster software were used to predict the functional effect of these mutations; the effects of the eight complement-related gene mutations on *MASP2, CFHR3, C8A, MASP2*, and *CFI* proteins were predicted to be damaging or probably damaging (Table 3).

rs12711521 in *MASP2* was identified in four patients; rs2273343 in *MASP2* was only identified in one patient. Four patients had mutations in more than two complement-related genes. Three mutations found in Patient 6 were also previously reported in patients with C3GN; therefore, these mutations were associated with an increased risk of C3GN [17]. Patient 2 possessed *C8A* and *MASP2* mutations; Patient 3 possessed *CFH* and *MASP2* mutations, and Patient 4 possessed *CFI* and *MASP2* mutations.

**Fig. 4:**
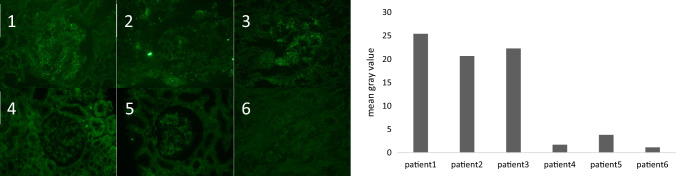
MASP2 Immunofluorescence staining: Patients 1, 3: fine granular global deposits (2 +) in capillary and mesangial area; Patient 2: fine granular global deposits (1 +) in capillary and mesangial area; Patients 4–6: negative. The right figure shows the mean gray value of MASP2 staining

We detected *MASP2* mutations in 5 atypical C3-PIGN patients. MASP2 is a key protein involved in the lectin pathway of complement. To further observe the activation of the lectin pathway, we stained for MASP2 expression in kidney biopsy tissue obtained from all patients. Patient 1 and patient 3 showed 2 + , and patient 2 showed 1 + positive deposition in mesangial areas and capillaries. The other patients were negative (Fig. 4).

**Fig. 5:**
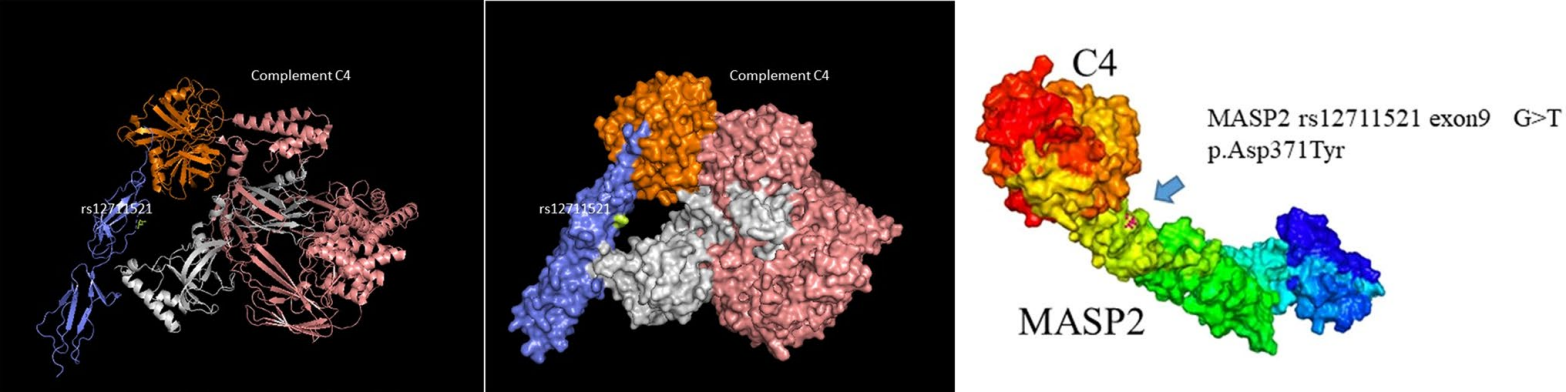
Three-dimensional models for the MASP2 protein was based on Protein Data Bank (PDB ID 5JPM) and Key amino acids with the GWAS association (rs12711521) is highlighted. Graphical representations of the 3D models were prepared using the PyMOL Molecular Graphics System, Version 2.5.2. MASP2 is shown in blue and white color, C4 is shown in orange and pink color. The rs12711521 (p.Asp371Tyr) is shown in green color and close to the binding site of MASP2 and C4

rs12711521 leads to the change of aspartic acid 371 to tyrosine (p.D371Y). We demonstrated that the 3D structure of MASP2 included rs12711521. We found that the mutated tyrosine 371 (green color) is located in the CCP2 domain, which is encoded by exon 9, and close to the binding site of C4, which may influence the secondary interaction with C4 (Fig. 5).

## Discussion

Most patients with C3-PIGN exhibit a relatively benign disease course, and hypocomplementemia is self-limiting. Approximately 8.8% of C3-PIGN patients exhibit persistent hypocomplementemia accompanied by abnormal urinary parameters; therefore, these atypical C3-PIGN patients need special attention. We found that atypical C3-PIGN patients had heterogeneous phenotypes and exhibited different ranges of microscopic hematuria and proteinuria. The duration of abnormalities in the levels of urinary markers also varied among these patients. Glucocorticoid therapy can significantly reduce the recovery time needed to resolve abnormalities in urinary test results and reverse hypocomplementemia, thus suggesting that immunosuppressive therapy may help to ameliorate the immune response in these patients.

The predominant histological changes associated with atypical C3-PIGN were proliferative mesangial lesions. Some patients have segmental proliferative lesions. In addition to subepithelial hump-like electron-dense deposits, mesangial, subendothelial and intramembranous deposits were also evident. Atypical C3-PIGN and C3GN share the similar immunofluorescence feature of C3-dominated deposition. Our observations indicated that it is difficult to make a differential diagnosis through histological changes. Clinical presentations, autoantibodies against complement components and the presence of gene mutations became important for distinguishing them. Typical PIGN patients with hematuria, proteinuria, and hypocomplementemia normally recover within 12 weeks, and renal function returns to normal. Therefore, in any patient with C3-PIGN, persistent clinical abnormalities, including hypocomplementemia, proteinuria or declining renal function, should lead to further investigation of autoantibodies and gene mutations associated with the complement system.

All of these atypical C3-PIGN patients had negative results in complement-related antibody testing. Persistent hypocomplementemia may be caused by the mutations of genes involved in the complement system. Previous studies demonstrated that gene mutations could be detected in ‘atypical PIGN’ patients with long-term abnormal urinary test results [18–20]. However, these reported gene mutations in ‘atypical PIGN’ patients with persistent abnormal urinary test results were all located in genes involved in the alternative pathway (AP), which was consistent with a decrease in serum C3 levels and the deposition of C3 in glomeruli, indicating abnormal activation of AP. Sethi et al. investigated mutations in the genes involved in the AP in 11 ‘atypical PIGN’ patients and found that four patients had mutations located in the CFH and CFHR5 genes [12]. Our atypical C3-PIGN patients also carried mutations in *CFH* and *CFHR5*. Additionally, two patients in our study had mutations in the *CFI* and *CFHR3* genes, which are also involved in the alternative pathway. One patient possessed mutations in *CFH, CFHR3*, and *CFHR5*. These mutations have been previously reported in C3GN patients and showed obvious linkage disequilibrium, thus increasing the susceptibility to kidney injury. Mechanistic studies confirmed that the mutation site in the *CFHR3* gene is located in the binding region of C3b, thus leading to a reduction in the binding affinity of CFHR3 and C3b [17], which may inhibit the inactivation and degradation of C3b.

Notably, five patients also possessed *MASP2* mutations; these mutations have also been described previously in C3G patients [21]. MASP2 is one of key serine proteases for complement activation in the lectin pathway (LP) and cleaves C2 and C4 to form the C3 convertase C4b2a. Activation of LP pathway plays an important role in IgA nephropathy and systemic lupus erythematosus. MASPs mediate process of coagulation and endothelial and platelet activation. Abnormal levels of MASP2 are associated with infections and inflammatory diseases, but the cases are rare and the clinical penetrance is also low.

In our study, we found four patients with the rs12711521 SNP variant. The tag SNP rs12711521 leads to the change of aspartic acid 371 to tyrosine (p. D371Y), and the predicted 3D structure of MASP2 with rs12711521 showed that tyrosine 371 is close to the serine protease domain which may influence the interaction with C4 and the stability of LP activation.. Interestingly, a previous study reported that the rs12711521 SNP is associated with high serum levels of MASP-2 [22] and susceptibility to numerous infectious diseases [23–25], and it is involved in dimerization [26] and increases MBL binding capacity [27]. These results suggested that patients carrying the rs12711521 risk allele are not only more susceptible to infection but also exhibit aberrant complement activation after infection. This speculation may explain why patient 1, who carried rs12711521, had suffered relapse when evaluated during the follow-up. We found that three patients with *MASP2* gene mutations exhibited MASP2 deposits in the glomeruli, which suggested that there may be complement activation of the lectin pathway in these patients [28]. The mechanisms underlying the role of *MASP2* mutations in the pathogenesis of C3-PIGN need to be further studied.

Expected genes in the alternative complement pathway and lectin pathway and *C8A* mutation were also found in one patient. *C8A* is a gene encoding the C8α subunit of complement and is involved in the formation of C5b-9. The polymorphism of *C8A* is related to Neisseria infection [29]. The majority of the mutations identified in the current patients were predicted to affect protein function when analyzed by SIFT or the Polyphen2 database and may induce abnormal immune responses to infections and cause persistently low serum levels of C3. Collectively, our results suggest that the continuous hypocomplementemia observed in atypical C3-PIGN patients is not only associated with abnormalities in the alternative complement pathway but also may be related to abnormalities in the lectin pathway and the pathway involved in resolving complement processes.

Unlike the gene mutations observed in C3GN patients, the gene mutations detected in atypical C3-PIGN patients may not lead to irreversible complement activation and subsequently severe renal damage. However, the existence of these mutations may induce delayed recovery of the complement system after infection and continuous hypocomplementemia. If stimulation is sustained, these atypical C3-PIGN patients may develop C3GN in the future. A previous publication reported that a second biopsy from patients with PIGN provided confirmation of C3GN [30]. Therefore, preventing repeated infections and continuous monitoring of the complement system are critical in the long-term management of atypical C3-PIGN.

A previous study demonstrated that C3GN patients possessing complement gene mutations may show familial aggregation of disease. The most typical familial disease in C3GN is CFHR5 nephropathy, a condition that has been reported in a family in Cyprus [31]. This condition is associated with autosomal dominant genetic characteristics; the clinical manifestations are continuous microscopic hematuria that may be accompanied by early onset pharyngitis and gross hematuria. Genetic testing showed that all members of this particular family possessed mutations in the *CFHR5* gene, which suggests that this may represent a disease involving a single gene. Other studies have reported that a series of mutations in the *CFHR* gene family were related to the phenomenon of familial aggregation [32, 33]. It is important to note that C3GN is a relatively complex genetic disease and is not often inherited in a Mendelian manner. We found no evidence of a positive family history of kidney disease in our present study.

Long-term follow-up led to the identification of one patient with persistent hypocomplementemia who had suffered relapses. The recurrence of PIGN is relatively rare. The recurrence of PIGN with immunoglobulin deposition has been suggested to be related to IgA deficiency [34]; mutations in *CFH* and *MCP* have been detected in patients with recurrent C3-PIGN [9]. We speculate that in the context of complement-related gene mutations, potential abnormalities in complement regulatory function are more likely to increase the possibility of relapse.

This study had some limitations. First, this was a retrospective study. We could not detect some antibodies, including C4 nephritis factor, C5 nephritis factor, Factor B, anti-C3b and anti-C1q, in these patients. Second, the function and pathological mechanisms of these identified abnormal mutations need further investigation.

In summary, we found that mutations in complement-related genes are relatively common in atypical C3-PIGN patients. The mutations are not only located in alternative complement pathway genes but are also found in genes involved in the lectin pathway and C5b9 combination. Although these patients exhibit a relatively benign disease course and glucocorticoid therapy could accelerate recovery time, atypical C3-PIGN patients with gene mutations exhibit abnormal complement activation reactions and delayed complement recovery periods. Sustained stimulation may cause the recurrence and aggravation of the disease and even lead to the progression to C3GN. Therefore, we emphasize the importance of detecting gene mutations, closely monitoring changes in the complement system, and providing active treatment for atypical C3-PIGN patients to improve long-term outcomes.
